# Acute COVID-19 Management in Heart Failure Patients: A Specific Setting Requiring Detailed Inpatient and Outpatient Hospital Care

**DOI:** 10.3390/biomedicines11030790

**Published:** 2023-03-06

**Authors:** Alberto Palazzuoli, Matteo Beltrami, Peter A. McCullough

**Affiliations:** 1 Cardiovascular Diseases Unit, Department of Medical Sciences, Le Scotte Hospital University of Siena Italy, 53100 Siena, Italy; 2 Cardiology Unit, San Giovanni di Dio Hospital, Azienda USL Toscana Centro, 50134 Florence, Italy; 3 Truth for Health Foundation, Tucson, AZ 85728, USA

**Keywords:** heart failure, COVID-19, treatment, cardiovascular events

## Abstract

The relationship existing between heart failure (HF) and COVID-19 remains questioned and poorly elucidated. Many reports suggest that HF events are reduced during pandemics, although other studies have demonstrated higher mortality and sudden death in patients affected by HF. Several vascular, thrombotic, and respiratory features may deteriorate stable HF patients; therefore, the infection may directly cause direct myocardial damage, leading to cardiac function deterioration. Another concern is related to the possibility that antiviral, anti-inflammatory, and corticosteroid agents commonly employed during acute COVID-19 infection may have potentially deleterious effects on the cardiovascular (CV) system. For these reasons, HF patients deserve specific management with a tailored approach in order to avoid arrhythmic complications and fluid retention events. In this review, we describe the complex interplay between COVID-19 and HF, the evolving trend of infection with related CV events, and the specific management strategy to adopt in this setting.

## 1. Introduction

The prevalence of new-onset heart failure (HF) or HF decompensation during COVID-19 infection remained questioned [1,2,3]. Some reports suggest a decrease in HF hospitalization events in subjects with a previous HF diagnosis [4]. Conversely, a diagnosis of HF is a recognized risk factor for a more severe clinical course during infection. This concept is still under scrutiny since HF populations are heterogeneous, often elderly, and mostly affected by heart failure with preserved ejection fraction (HFpEF) with many concomitant non-cardiovascular comorbidities, conferring a high-risk profile irrespective of HF diagnosis. Additionally, COVID-19 infection is an important trigger for de novo HF as a consequence of myocardial injury or other major cardiovascular complications or for an acute decompensation of chronic HF.

COVID-19 significantly impacts the outcome in patients with or without left ventricular (LV) systolic dysfunction. A retrospective study demonstrates a higher incidence of COVID-19-related hospitalization or death in patients with pre-existing left ventricular ejection fraction (LVEF) < 40% and submitted to percutaneous coronary intervention (PCI) [5]. In a cohort of 692 patients in Italy, mortality was higher in those with pre-existing HF compared to those without HF, even after adjustment for confounding factors [2]. Therefore, HF patients hospitalized for COVID-19 infection show a higher rate of hospital complications, such as acute kidney injury, sepsis, and multi-organ failure [6]. Some results were demonstrated in a group of male veterans in the USA, where 30-day mortality was higher in those with LVEF < 45% [7]. Lassen et al. found a significantly reduced global longitudinal strain (GLS), LV diastolic function, and right ventricular (RV) function in COVID-19 cases compared to controls with a higher rate of death in patients with LV/RV systolic dysfunction [8]. International societies have recently published reports focused on the management of patients with COVID-19 and HF based on the available data and personal experiences of physicians from the USA, Europe, and Asia [9,10]. Many gaps in knowledge remain to be clarified about the effective mechanisms involved in cardiac function deterioration during COVID-19 infection, the relationship between pre-existing HF diagnosis and outcome, and specific management for patients affected by HF to be applied in an acute setting or during convalescence periods [11]. 

## 2. Impact of COVID on the Stable HF Population

The pandemic changes HF healthcare services, including outpatient clinics and home visits, with early evidence of worse long-term outcomes [12]. Hospitals and out-patient clinics canceled cardiology visits and elective surgeries, as well as important diagnostic investigations [13]. In a significant cohort of outpatients with HF patients, as determined by a telephone interview, COVID-19 was associated with increased psychological stress, worsening lifestyle patterns such as tobacco use, weight gain, and a reduction in physical activity. Such modifications increase cholesterol levels, blood pressure, and insulin resistance, with a consequent CV risk elevation. In the UK, a telephone questionnaire reported higher anxiety levels regarding COVID-19 than HF, as well as a reluctance to attend hospital visits and medication provision services [14]. The strategies to reduce the spread of the infection led to a significant limitation of elective face-to-face appointments, and many patients did not have their HF treatment optimized with consequent clinical deterioration and HF hospitalization. All of this led to sub-optimal management, delayed diagnosis, and preventive/early treatment of new HF cases, with a substantial increase in the rate of adverse fatal CV events.

**Figure 1:**
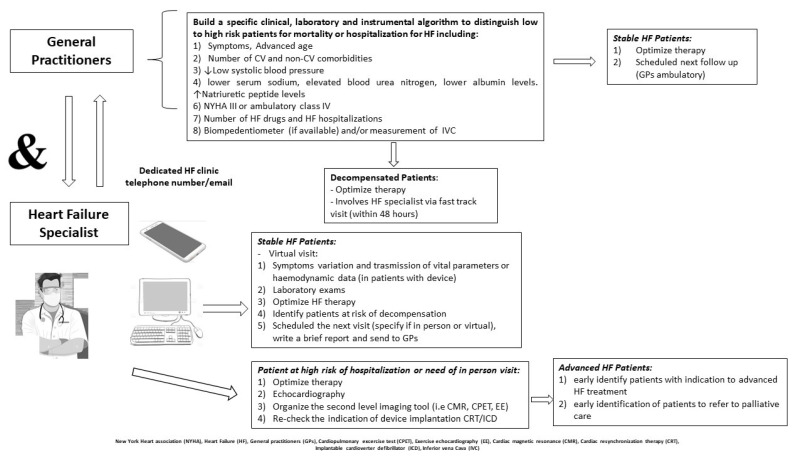
Cross-discussions between the General Practitioner and the Heart Failure specialist: check-up schedule may be planned in relation to HF symptoms and the occurrence of CV and non-CV diseases.

The nature of the pandemic has driven the need to facilitate initiatives promoting patient education, the prescription and optimization of appropriate drug therapies, and post-discharge organization. Nowadays, the COVID-19 pandemic seems to be moving slowly towards an endemic situation, and there is an unmet need to learn how HF clinics have reorganized HF care in various countries with different infection levels, vaccination rates, and healthcare opportunities to avoid unnecessary infections. Novel strategies to deal with patients remotely, such as video consultations and home telemonitoring, may result in more regular contact and ultimately benefit patients living with HF. In particular, central registration of some easily measurable variables such as heart rate, blood pressure, oxygen saturation, and body weight could detect the prodromal signs of HF and permit treatment optimization while avoiding HF hospitalization [15] (Figure 1). Recent experience in New York with a multisensor device may suggest the beneficial effects of the current approach in reducing both HF hospitalization and COVID-19 infection [16]. Furthermore, many patients, faced with restrictions, have taken more ownership of their health and well-being, and this can only be a good thing in a wider sense.

## 3. Difference in Cardiovascular Comorbidities, Cardiovascular Complications, and Survival across the Pandemic Waves

New treatment options, in-hospital and health care system experience, less aggressive COVID-19 variants, and the commencement of vaccination led to a substantial change in cardiovascular-related complications and mortality in patients with HF and/or CV disease across the epidemic waves of COVID-19 infection. Additionally, some gaps in knowledge regarding initial confusion in management have been filled and improved: First, guidelines were introduced and adapted for the dosage of low-molecular-weight heparin thromboprophylaxis in COVID-19, resulting in a substantial reduction of thrombotic complications [17]. Furthermore, Remdesivir, which halved viral production, led to a lower risk of hospitalization, a shorter hospital stay, or death, with an overall reduction of infection-related detrimental cardiovascular events. Dexamethasone demonstrated to reduce mortality in critically ill COVID-19 patients, and recent data highlighted the clear reduction of in-hospital intensive care hospitalization for HF patients with a history of vaccination during the SARS-CoV-2 Omicron wave, which was more pronounced in those with a booster dose [18]. An interesting analysis from the MedStar Health system compared the clinical characteristics and outcomes in patients with myocardial injury across the COVID-19 waves. Baseline characteristics and troponin levels were similar overall. The use of remdesivir and dexamethasone was highest in the second and third waves. During the third wave, half of the population enrolled was vaccinated. In-hospital mortality, intensive care unit (ICU) admissions, and decreased mechanical ventilation were significantly lower during the third wave compared to earlier waves of the pandemic; patients who were vaccinated showed more favorable in-hospital outcomes than did those who were unvaccinated [19]. The most striking feature observed in the COVID-19 infection was the abnormal hypercoagulative status, which is associated with high incidences of thrombotic complications. SARS-CoV-2 can trigger a cytokine storm that ultimately leads to the activation of the coagulation cascade. The Dutch COVID-19 and Thrombosis Coalition revealed a substantial mortality reduction between the first and second waves, although the thrombotic complications rate remained high and comparable to the first wave [20]. These data suggest that despite similar thrombotic events, early anthitrombotic/anti-inflammatory and antiviral management can decrease the overall mortality.

In the UK, patients with HF had comparable clinical characteristics, comorbidities, cardiovascular risk factors, and mortality during the first three waves, although medical therapy improved with a higher proportion of patients receiving angiotensin receptor-neprilysin inhibitors in the second and third lockdowns [21]. In the Japanese nationwide registry, patients hospitalized during the first COVID-19 wave had a longer hospital stay with higher cardiovascular complications. Patients hospitalized during the second and fifth COVID-19 waves had a lower mean age than those hospitalized during the other COVID-19 waves. In the fifth COVID-19 wave, patients exhibited a greater number of presenting symptoms, a higher percentage of patients required oxygen therapy at the time of admission, and a higher incidence of invasive mechanical ventilation. The mortality rate was the highest in the third COVID-19 wave. Patients with prior cardiovascular disease were respectively 7.8% in the first wave, 9.4% in the second wave, 11.5% in the third wave, 11.3% in the fourth wave, and 7.8% in the fifth wave [22]. Current data should not be viewed in isolation, but rather in relation to underlying CV risk factors and diseases. Notably, an interesting Italian cohort study reporting baseline conditions showed that during the first wave, hypertensive heart disease was mentioned as a comorbidity in 18.5% of death certificates, followed by diabetes and ischemic heart disease. Neoplasms were the most common comorbidity during the second wave, followed by hypertensive heart disease and diabetes [23]. In the same country, hospitalization during the second wave and third wave was associated with a reduced risk of COVID-19 death in comparison with the first wave; the prevalence of patients with heart disease at admission was 48.5%, 58.1%, and 58.4%, respectively, for each wave; nevertheless, there was no difference in survival probability in patients aged >75 years [24]. In a Brazilian population, chronic cardiovascular disease was more prevalent in the first and second waves of COVID-19 deaths; nevertheless, deaths were more frequently associated with chronic cardiovascular disease in the third wave, maybe related to sicker patients with advanced cardiovascular disease [25]. Similar results were found in three Canadian provinces, where the total burden of cardiovascular disease was higher during the first and second waves, with a significant reduction in total mortality and the need for invasive mechanical ventilation, vasopressors, and renal replacement therapy in patients with HF during the third wave [26]. In a Northeastern France population, baseline characteristics of hospitalized patients during the first and second waves did not differ in terms of age, sex, BMI, or cardiovascular comorbidities, but admission to the intensive care unit and deaths significantly decreased during the second wave. A possible explanation was the positive correlation with disease severity, with more severe patients being at higher risk of cardiovascular complications [27]. The cumulative incidence of COVID-19 hospitalization and mortality, according to the whole population, increased from wave 1 to wave 2 in patients with diabetes. However, when mortality was related only to the hospitalized patients, the crude mortality was significantly reduced in all the diabetic patients [28]. During the five waves of the COVID-19 pandemic in Romania, Fericean et al. found substantial changes in overweight and obese patient features. Obesity and obesity-related comorbidities increased the risk of hospitalization, severe complications, and mortality across all the waves; moreover, during the 4th wave, obese patients showed higher proportions of ICU admissions and cardiovascular mortality [29]. These findings suggest that while the prevalence of thrombotic complications and myocardial damage remained similar across different waves and virus variations, early tailored treatment is capable of reducing both CV complications and mortality.

## 4. Care of Heart Failure Patients with COVID-19

### 4.1. HF Gaps in COVID-19

The recent position paper addressed HF care and was mainly focused on the adverse pro-arrhythmic effects of antiviral and anti-inflammatory drugs commonly employed in infected patients and the potential effects of ACE inhibitors and betablockers on virus diffusion in endothelial and epithelial cells with consequent CV and respiratory system deterioration. This concern has been recently elucidated by some evidence showing a protective effect of renin-angiotensin-aldosterone system inhibitors (RAASi) [30,31]. Another paper debated the correct application of more aggressive devices in patients with multiorgan failure and advanced HF as well as the possible advantages of managing these patients at home by using telemonitoring opportunities [32]. However, some issues regarding the concomitant use of anti-arrhythmic drugs during infection, the related ECG screening before and during antiviral therapy, and the effects of corticosteroid administration on the metabolic and CV systems have been poorly reported. 

### 4.2. Treatment Effects of Current Antiviral Treatments

There are no large conclusive randomized double-blind placebo-controlled trials of any antiviral in the treatment of COVID-19. Such a trial would need to include >20,000 patients and have the power to detect a significant reduction in hospitalization/death. Smaller, unblinded trials have not been conclusive. Thus, we must consider drugs with a signal of benefit and acceptable safety. The Table lists available drugs and summarizes the evidence for the two most widely available antivirals (hydroxycholorquine and ivermection), both of which are featured in dozens of national and international guidelines around the world (Table 1). Therefore, a higher amount seems to be associated with an increase in the rate of adverse events and arrhythmic complications [33,34]. Despite the few cases of sudden death directly related to anti-inflammatory drugs that have been described, an increase in the QT interval predisposing to arrhythmia is a common finding revealed during treatment. Current prolongation is universally considered dangerous if the QT interval is >450 msec. However, no studies have deeply investigated which patients are prone to develop QT prolongation, the weight of amplifying effects of electrolyte disorders, cytokine storms, and the acidosis status often associated with sepsis [35]. Therefore, anti-inflammatory agents are usually administered together with macrolides and antiviral drugs (i.e., remdesivir) that could impair hepatic function, reducing both drug excretion and drug-kinetic activity, with potential symbiotic effects on QT prolongation [36,37]. Current conditions may exacerbate ECG alterations, reducing the arrhythmic threshold, and they require careful ECG monitoring during active treatment with a weekly check for outpatients and daily control for hospitalized subjects with more severe respiratory conditions [38]. Since further systemic and metabolic causes may potentially induce arrhythmia, the ECG monitoring might be combined with electrolyte, inflammatory indexes, specific cytokine assays, and blood gas analyses to verify potential contributing features. 

Baricitinib is the last antiviral drug with a double effect on SARS-CoV2 infection: it reduces the inflammatory response through the inhibition of the Janus-Kinase signaling transducer and activator of transcription (JAK-STAT) pathway. Moreover, it acts on AP2-associated protein kinase 1 (AAK1) inhibition, reducing viral endocytosis [39]. In theory, the two effects may reduce cardiovascular events, but the increased prothrombotic risk in COVID-19 requires more studies, especially in patients with altered coagulation [40]. Regarding anti-interleukin agents tocilizumab, anakinra, baricitinib, and monoclonal antibodies (bamlanivimab, etesevimab, and casirivimab), no evidence supports significant effects on cardiac ion channels, changes in the QT interval, or increased risk for ventricular arrhythmias [41]. Additionally, anakinra demonstrates potential beneficial effects in patients with myocarditis-related infection by reducing the inflammatory and immune modulated response, providing a consistent reduction in myocardial oedema and associated pericardial effusion [40]. IL-1β is a proinflammatory cytokine that is involved in the autoinflammatory pathogenesis of the COVID-19 infection. Canakinumab, an anti–interleukin-1β antibody, has been used against COVID-19 infection without significantly increasing the likelihood of survival in patients with severe COVID-19 infection [41].

### 4.3. The Role of Anti-Arrhythmic Agents

Many patients with HF are usually treated with anti-arrhythmic drugs (particularly class III) to prevent or reduce supraventricular or ventricular arrhythmias. Specific categories with an implantable cardioverter defibrillator device or those at high risk for ventricular tachycardia taking amiodarone or sotalol might be considered at higher risk. Both drugs are capable of prolonging the QT interval and during particular conditions such as metabolic acidosis or sudden K^+^, Ca^+^, or Mg^+^ reduction, they might decrease the arrhythmic threshold with unfavorable effects on the cardiac electrical system. Current features during septic status need to be accounted for “per se” as favorable conditions for an arrhythmic storm. In the context of concomitant use of antiviral, anti-inflammatory, or macrolide treatments, the probability of an arrhythmic event is further amplified. Thus, the combined use of antiarrhythmic and COVID-19 treatment may be carefully customized or interrupted whether the ECG demonstrated an increase rate of ectopic ventricular beats or evidence for significant QT lengthening [42]. Up until now, no randomized studies have investigated this aspect, but even in the absence of specific data, a caution for the contemporary use of antiviral and antiarrhythmic agents may be warranted. 

### 4.4. Corticosteroids, Diuretic Agents and SGLT-2 Inibhitors

Recent analysis reported a beneficial effect of dexamethasone therapy in reducing deleterious immune responses against virus antigen [43]. The main caveat with dexamethasone as an unconventional steroid is underdosing. Thus, methylprednisone is commonly used in standard ICU or inpatient dosing. Likewise, prednisone is deployed in outpatient care. A positive anti-inflammatory effect with a decrease in plasma cytokine levels and infection remission have been observed in both hospitalized and non-hospitalized subjects [44]. After these observations, there are a few ongoing protocols aimed at verifying the preliminary findings. Whether current results will be confirmed, several concerns about the potential deleterious effects of corticosteroid therapy on the CV, metabolic, and capillary systems might be accounted for [45]. Indeed, dexamethasone increases fluid and sodium retention, enhances peripheral vasoconstriction, and increases cardiac workload. Such effects have unavoidable consequences on blood pressure values, idro-saline retention, and body fluid shift from the intravascular district to the interstitium. Current mechanisms could become potential triggers for HF onset or recurrence, and they deserve careful monitoring through a systematic check of blood pressure, body weight, and diuresis [46]. Behind CV manifestations, other metabolic effects could be related to chronic corticosteroid therapy: altered glycemic control, derangement of the arginine vasopressin axis, and ACTH-cortisol feedback may occur during repetitive administration. All these aspects cannot be ignored, particularly in patients with HF stages A and B and those with initial NYHA classes, in whom corticosteroid therapy may worsen HF status. Therefore, a diuretic dose titration during corticosteroid treatment could be advised to prevent systemic congestion and an increase in LV filling pressure. Specifically, loop diuretics may be targeted in relation to the dose of corticosteroids prescribed, the treatment duration, and the signs of congestion [47,48] (Table 1).

Recent research demonstrated that SGLT-2 inhibitors are associated with a significant decrease in the inflammatory pathway, reducing, for example, C-reactive protein, interleukins, and ferritin. Moreover, SGLT-2 inhibitors lead to a benefit in vascular endothelium function with a sort of protection against the thrombotic issues of COVID-19 [49]. A recent meta-analysis showed that early use of SGLT-2 inhibitors as glucose-lowering treatment may reduce the mortality risk and severity of COVID-19, independently from age, sex, and comorbidities [50]. The DARE-19 trial demonstrated the safety and efficacy of dapagliflozin on cardiovascular death and renal outcome in hospitalized patients with COVID-19. The results of the CHIEF-HF study confirmed the benefits of canagliflozin in improving patients’ symptom burden, both in patients with HFrEF and HFpEF [51].

## 5. Community Standard of Care for Outpatient COVID-19 in High-Risk Patients

**Figure 2:**
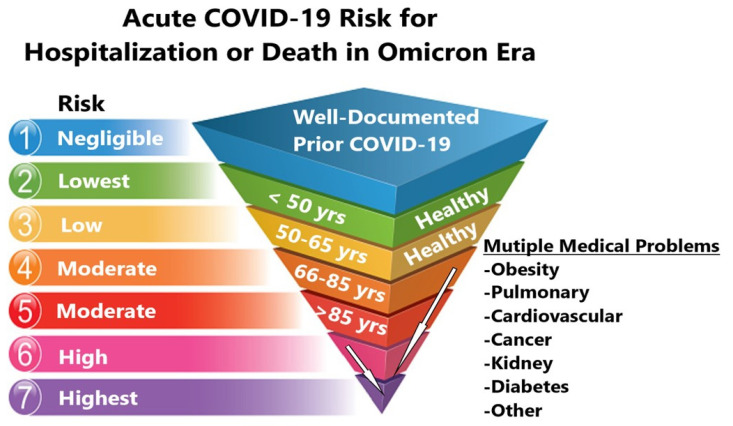
High-risk patients for Heart Failure hospitalization and/or cardiovascular disease.

**Figure 3:**
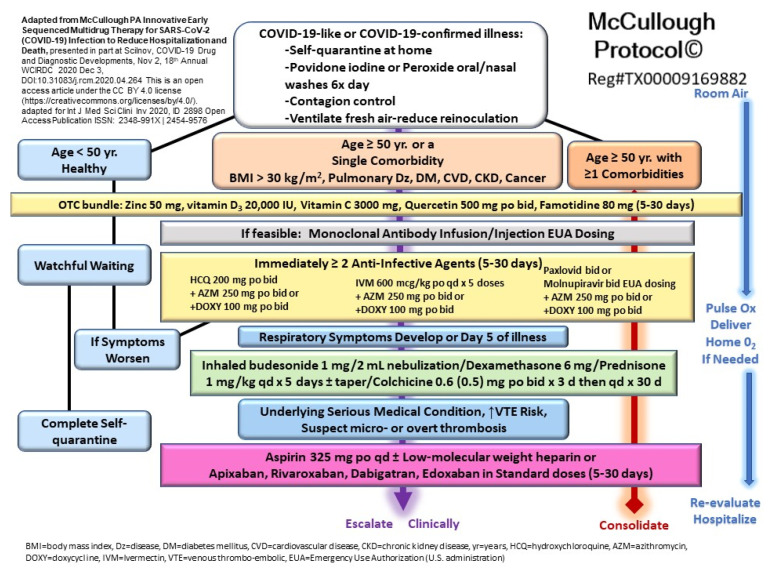
The McCullough protocol for the COVID-19 home treatment guide.

Outpatient management is the community standard of care and should be given to all high-risk patients with acute COVID-19 on the index episode (Figure 2). Subsequent episodes of COVID-19 are characteristically mild and do not require intensive pharmacotherapy [52]. The McCullough protocol as shown is the basis for community standard of care as published in the Association of American and Physicians home treatment guide and is shown in the protocol (Figure 3, aapsonline.org) [53]. Treatment starts with nasal washes and gargles with dilute povidone iodine (<1%) or dilute hydrogen peroxide every 4–6 h. Twelve clinical studies and three large RCTs demonstrate reductions in viral load and risk of hospitalization or death. Over-the-counter neutraceutals and medications are modestly helpful and supported by published studies, and the list includes: zinc, vitamin C, vitamin D, quercetin, full-dose aspirin, and famotidine. These should be in the house and ready to be used on the first day of illness. For very high-risk individuals (class III HF, nursing home patients), intravenous monoclonal antibodies can be used as a single-dose infusion in the first few days of illness [54]. Next is the use of oral antivirals which can be combined with antibiotics if there is a risk of superimposed atypical bacterial organisms or bacterial bronchitis. Oral colchicine should be used for 30 days. Inhaled and oral corticosteroids can be deployed on day three or beyond based on symptoms. Lastly, for bed-bound patients or those at high risk for thromboembolic complications, parenteral low molecular weight heparin or oral anticoagulants can be deployed for 30–90 days [55]. In summary, use of 4–6 drugs in a sequenced combination has been associated with substantial reductions (>85%) in hospitalizations and deaths. By December 2020, the evidence that early therapeutic protocols reduced these hard endpoints was clear and convincing (*p* < 0.01) [56]. Conversely, studies of hospitalized patients demonstrated a lack of early treatment as an obvious determinant of hospitalization [57,58].

## 6. Conclusions

The interplay between COVID-19 infection and HF remains complex because infection may deteriorate a stable HF condition and concurrent events may promote vascular and respiratory complications leading to clinical deterioration. Additionally, some drugs tested in the acute phase of infection may facilitate arrhythmic events and fluid retention, leading to HF. High-risk patients with multiple CV diseases and an early HF stage deserve a careful approach, possibly avoiding the deleterious effects of some agents currently employed during hospitalization. In certain categories without respiratory complications, home care based on nutraceutical agents and common anti-inflammatory drugs is probably preferable in order to reduce hospitalization and guarantee tailored HF management.

## Figures and Tables

**Table 1:** Mechanism of action and cardiovascular side effects of common drugs used in COVID-19 patients.

Drugs	Mechanism of Action	Cardiovascular Side Effects
Aminoquinoline		
Hydroxychloroquine/chloroquine	-Interfere with lysosomal activity and autophagy, altering the PH of the lysosomes reduces low affinity self-antigen presentation. -Inhibits terminal glycosylation of ACE2 in SARS-COV-2.	-Conduction abnormalities (bundle branch block, atrioventricular block) -QTc prolongation -Hypoglycemia (mostly in diabetic patients)
Ivermectin	-Interfere with Spike protein attachment to RBCs -Block nuclear entry of SARS-CoV-2 -Modulate intracellular messengers of inflammation	None
Antiviral drug nucleoside analogue		
Remdesevir	Remdesevir is a phosphoramidate prodrug that contains an active nucleoside triphosphate and inhibits the RNA-dependent RNA polymerase of coronaviruses.	-Sinus bradycardia -QTc prolongation and torsade de point -AF trigger -T-wave abnormalities -Cardiac arrest -Significantly increased risk of acute kidney injury
Janus kinase inhibitor		
Baricitinib	-Reduces the inflammatory response through the inhibition of the Janus-Kinase signaling transducer and activator of transcription pathway. -It acts on AP2-associated protein kinase 1 inhibition, reducing viral endocytosis.	- Thrombosis, including deep venous thrombosis and pulmonary embolism -Higher rate of major adverse cardiovascular events (cardiovascular death, myocardial infarction, and stroke) compared to the placebo
Corticosteroids		
Dexamethasone	-Inhibits the pro-inflammatory pathway that encodes for chemokines, cytokines, cell adhesion molecules, and the acute inflammatory activation in response to SARS-CoV-2 infection.	-Elevations of total plasma cholesterol and triglycerides -Abnormal glycemic control -Increased systolic blood pressure and weight -Fluid and sodium retention, HF occurrence
Interleukin antagonists		
Anakinra	-Is a recombinant, non-glycosylated form of the human IL-1 receptor antagonist that binds the IL-1 receptor, reducing the inflammatory response.	Not described
Interleukin-6 inhibitors		
Tolicizumab	Tocilizumab and sarilumab are both IL-6 receptor antagonists that prevent the downstream activation of IL-6.	-Hypertension -Increased levels of cholesterol and/or triglycerides
Sarilumab		-Cardiac failure -Embolic and thrombotic event
Interleukin-1 inhibitors		
Canakinumab	It is a human IgG1k monoclonal antibody that neutralizes soluble IL-1β.	Not described
Monoclonal Antibodies		
Etesevimab	Monoclonal antibodies specifically bind the virus’ surface spike protein receptor binding domain. This high affinity is related to the strong binding of the ACE2 host cell surface receptor.	-Hypertension
Bamlanivimab		-Hypertension -Ischemic heart disease
Casirivimab		-Hypertension -Ischemic heart disease

Angiotensin converting enzyme 2 (ACE2), Heart Failure (HF), Atrial Fibrillation (AF), Interleukin-1 receptor (IL-1R).

## Data Availability

Not applicable.
